# Exploring fungal biodiversity: organic acid production by 66 strains of filamentous fungi

**DOI:** 10.1186/s40694-014-0001-z

**Published:** 2014-11-01

**Authors:** Nadège Liaud, Christian Giniés, David Navarro, Nicolas Fabre, Sylvaine Crapart, Isabelle Herpoël- Gimbert, Anthony Levasseur, Sana Raouche, Jean-Claude Sigoillot

**Affiliations:** 1 INRA, UMR1163 Biotechnology of Filamentous Fungi, Marseille, F-13288 France; 2 Aix Marseille Université, UMR1163 Biotechnology of Filamentous Fungi, Marseille, F-13288 France; 3 ARD, Agro-Industry Research and Development, Pômacle, F-51100 France; 4 INRA, UMR 1260, « Nutrition, Obésité et Risque Thrombotique », Marseille, F-13385 France; 5 INSERM, UMR 1062, « Nutrition, Obésité et Risque Thrombotique », Marseille, F-13385 France; 6 Université d’Aix-Marseille, UMR 1260, « Nutrition, Obésité et Risque Thrombotique », Faculté de Médecine, Marseille, F-13385 France; 7 INRA, International Center for Microbial Resources collection-Filamentous fungi CIRM-CF, Marseille, F-13288 France; 8 Polytech’ Marseille (ex ESIL), UMR 1163 BCF - INRA / AMU, 163 Avenue de Luminy CP 925, Marseille, F-13288 France

**Keywords:** Low molecular weight organic acids, Ethanol, Filamentous fungi, Ascomycota, Basidiomycota, Biodiversity

## Abstract

**Background:**

Filamentous fungi are well known for their ability to degrade lignocellulosic biomass and have a natural ability to convert certain products of biomass degradation, for example glucose, into various organic acids. Organic acids are suggested to give a competitive advantage to filamentous fungi over other organisms by decreasing the ambient pH. They also have an impact on the ecosystem by enhancing weathering and metal detoxification. Commercially, organic acids can be used as chemical intermediates or as synthons for the production of biodegradable polymers which could replace petroleum-based or synthetic chemicals. One of the advantages of filamentous fungi as biotechnological production platforms for synthetic biology is their ability to degrade vegetal biomass, which is a promising feedstock for the biotechnological production of organic acids. The Fungal Culture Collection of the International Centre of Microbial Resources (CIRM-CF), curated by our laboratory, contains more than 1600 strains of filamentous fungi, mainly *Basidiomycetes* and *Ascomycetes*. The natural biodiversity found in this collection is wide, with strains collected from around the world in different climatic conditions. This collection is mainly studied to unravel the arsenal of secreted lignocellulolytic enzymes available to the fungi in order to enhance biomass degradation. While the fungal biodiversity is a tremendous reservoir for “green” molecules production, its potentiality for organic acids production is not completely known.

**Results:**

In this study, we screened 40 strains of *Ascomycota* and 26 strains of *Basidiomycota,* representing the distribution of fungal diversity of the CIRM-CF collection, in order to evaluate their potential for organic acid and ethanol production, in a glucose liquid medium. We observed that most of the filamentous fungi are able to grow and acidify the medium. We were also able to discriminate two groups of filamentous fungi considering their organic acid production at day 6 of incubation. This first group represented fungi co-producing a wide variety of organic acids and ethanol at concentrations up to 4 g.L^−1^ and was composed of all the *Aspergilli* and only 3 other *Ascomycota*. The second group was composed of the remaining *Ascomycota* and all the *Basidiomycota* which produced mainly ethanol. Among the *Basidiomycota,* two strains produced oxalic acid and one strain produced gluconic and formic acid. Six strains of *Aspergillus* producing high concentrations of oxalic, citric and gluconic acids, and ethanol were selected for metabolism analysis.

**Conclusion:**

These results illustrate the versatility in metabolites production among the fungal kingdom. Moreover, we found that some of the studied strains have good predispositions to produce valuable molecules. These strains could be of great interest in the study of metabolism and may represent new models for synthetic biology or consolidated bioprocessing of biomass.

**Electronic supplementary material:**

The online version of this article (doi:10.1186/s40694-014-0001-z) contains supplementary material, which is available to authorized users.

## Background

Low molecular weight organic acids production by filamentous fungi, have attracted considerable attention for their role in natural ecology and their potential industrial applications [1],[2]. Fungal natural production of organic acids is thought to have many key roles in nature depending on the type of fungi producing them. These roles are either due to the pH decrease consecutive to their secretion or to direct interaction of the organic acid with the environment [3],[4]. The consecutive decrease in pH upon their secretion may give a competitive advantage to the acid-tolerant filamentous fungi. For ectomycorrhizal fungi, this pH decrease also has been suggested to solubilize soil minerals thus releasing nutrient ions for uptake by plants and microorganisms, enhancing mineral weathering [1]. For saprophytic and wood-decaying fungi, this pH acidification, caused by oxalic acid production, leads to an acid-catalyzed hydrolysis of holocellulose [5]–[7]. Concerning their direct interaction with the environment, organic acids participate in metal detoxification by metal complexion and oxalic acid plays a major role in biomass degradation [4]. For this reason, *Basidiomycota* have been extensively studied for their ability to produce oxalic acid [8]–[12]. To better understand their role in the ecosystem, these studies have focused on plant/fungi symbiosis [9],[13], or growth on complex substrates [12],[14]–[16], and are often focused on wood-decay or mycorrhizal fungal species.

In addressing the demand for sustainable alternatives to fossil fuel as a source of energy and chemicals, synthetic biology focuses on understanding how biological systems work and how to use them to benefit society. Organic acids can have multiple industrial applications as food additives, pharmaceutical and cosmetic excipients [17]. They are fully degradable molecules and can be used as chemical intermediates or as synthons for the production of biodegradable polymers, potentially replacing petroleum-based or synthetic chemicals [17]. Some fungi are well known for their natural capability to produce high amounts of various useful organic acids. These fungi are mostly from the *Aspergillus* (e.g. citric, gluconic, malic and itaconic acids) and *Rhizopus* genera (e.g. lactic and fumaric acids). Some of these organic acids (i.e. citric acid) can be produced through large-scale bioprocesses, showing the high potential of fungi as organic acid production plateforms [2],[18].

The literature concerning organic acid production in filamentous fungi often focuses on one specific organic acid and there is little information about the other metabolite produced. In many cases these studies focus on specific strains and cultures are carried out in different conditions and with different complex media. Therefore, it is sometimes difficult to compare the potentiality of filamentous fungi from the literature. Moreover, the fungal biodiversity is estimated to be 1.5 million species [19] and there is still a lot to learn about their potential for metabolite production. In this study, 66 strains of saprophytic and wood-decay fungi (40 *Ascomycota* and 26 *Basidiomycota*) were selected and studied in liquid glucose medium, without pH regulation, in order to compare their metabolic features. These strains belong to 47 different species, representing 23 fungal families. The majority of the strains tested were collected *in situ* from different geographical areas such as tropical forests from French overseas territories and temperate forests from metropolitan France [19]. Fungal growth and metabolites production was done in glucose liquid media without any pH regulation to take into account industrial up- and down-stream technical and economical issues. These conditions, close to industrial ones, were chosen to highlight the potential of these organisms for industrial organic acid production. The great diversity and origin of the selected strains enable us to compare the potentiality of a number of fungal groups for the production of organic acids and ethanol.

## Results and discussion

### Growth of the selected strains and pH of the medium

**Figure 1:**
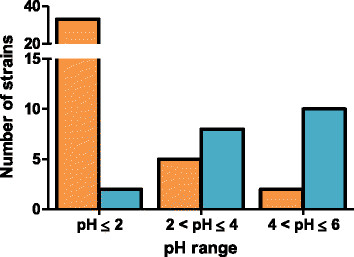
**Repartition of** ***Ascomycota*** **and** ***Basidiomycota*** **strains according to the final pH of growth medium after 6 days of incubation.** (orange square) *Ascomycota*, (sky blue square) *Basidiomycota*.

All the *Ascomycota* were able to grow in the liquid medium at an initial pH of 5.5. However, 4 strains of *Basidiomycota*: *Ischoderma benzoinum* (BRFM1133), *Grifola frondosa* (BRFM1162), *Panellus serotinus* (BRFM1284), and *Polyporus squamosus* (BRFM1531), did not grow to a sufficient level and did not acidify the medium. These strains were not considered for the following steps. The pH of the medium was acidified for most of the cultures but to different extents. An extreme acidification, to pH below or equal to 2, was observed for 33 *Ascomycota* strains, representing 80% of the strains from this phylum. In particular, all the *Aspergilli* tested (22 strains) acidified the medium below pH 2 (Figure 1). Five strains of *Ascomycota* acidified the medium to pH between 2 and 4 and only two strains did not or slightly acidify the medium. To the contrary, only two *Basidiomycota* highly acidified the medium to pH below or equal to 2, namely *Phanerochaete chrysosporium* (BRFM413) and *Trametes menziesii* (BRFM1281). Eight strains acidified the medium between 2 and 4, and 12 strains did not or slightly acidified the medium (Figure 1). For the strains acidifying the medium, the acidification started within 24 hours of growth and the final pH was already observed after 3 days of growth.

### Organic acid and ethanol production

**Figure 2:**
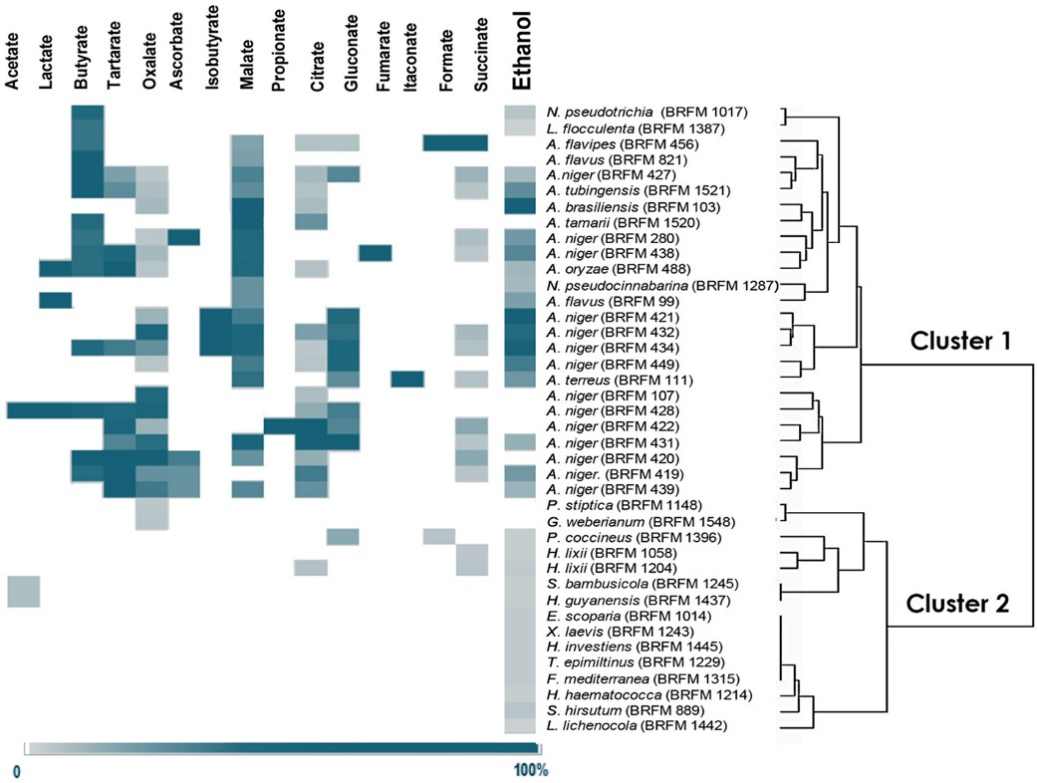
**Hierarchical clustering of organic acids and/or ethanol producing strains.** Concentration were determined by HPLC-UV or RI analysis and expressed as a percentage of the maximum concentration observed for each metabolite and represented by a color scale with different intensity of blue. Concentration of butyric, tartaric, oxalic, malic, citric, gluconic, succinic acids and ethanol were used to build distance tree. The figure was edited using the Multiexperiment Viewer software [20].

HPLC analysis of the supernatants obtained at day 3 of incubation contained only oxalic, malic, propionic and citric acids, found mostly in the supernatants of *Aspergillus* species. The samples taken at day 6 of incubation showed a better view of the potentiality of the strains tested for organic acid production and allowed the detection of 15 different carboxylic acids at concentration between 0.1 and 3.7 g.L^−1^: acetic, ascorbic, butyric, citric, fumaric, formic, oxalic, gluconic, itaconic, isobutyric, lactic, malic, propionic, succinic, and tartaric acids (Figure 2). Ethanol was the main neutral metabolite.

Hierarchical clustering was used to classify the strains producing detectable amounts of organic acids and/or ethanol at day 6 of incubation. The clustering was based on production levels of the most widely detected compounds: butyric, citric, gluconic, malic, oxalic, succinic, tartaric acids and ethanol (Figure 2). Two main groups appeared in this clustering: one group of organic acid and ethanol producers and one group producing mainly ethanol.

In the first group, which was mainly composed of *Aspergilli*, all the compounds analyzed in our assays were detected. This first group represented fungi co-producing a wide variety of organic acids at relatively high concentrations. Two sub-groups could be observed. The first one was composed of various *Aspergilli* and *Nectria* species, with only three *A. niger* species. The second one was composed mostly of *A. niger* species, with the exception of *A. terreus* (BRFM111). These results show that *A. niger* is clearly an exception in the fungal kingdom concerning organic acid production. Organic acid production may also be used along with secondary metabolites in the chemotaxonomy of *Aspergilli* [21].

The second group was composed of the remaining *Ascomycota* and *Basidiomycota* producing mainly ethanol. The metabolite concentrations obtained were lower than for the first group and the variety was narrower since only 6 different organic acids out of 15 were detected (Figure 2). In this second group, two subgroups were observed with strains producing only ethanol and strains producing ethanol and/or other organic acids. Ethanol was detected in 33 strains out of 40 regardless their phylum and species. Although this ethanol production is surprising for organisms traditionally considered as non-fermentative, there are previous records of ethanol production by filamentous fungi. Recent literature shows an increasing interest in ethanol production by filamentous fungi, in particular *Flammulina velutipes* [22],[23]. Some species belonging to *Fusarium*, *Mucor* and *Paecilomyces* were also found to efficiently convert xylose to ethanol with high yields [24]-[26].

Concerning the relation between pH acidification (section Growth of the selected strains and pH of the medium) and organic acid production, as expected most of the highly acidifying strains were good organic acid producers, from the *Ascomycota* phylum. However, the pH obtained in the *Ascomycota* growth media was below 2, which is far below the pKa of organic acids (between 3 and 5). Moreover, some strains for example *Phanerochaete chrysosporium* (BRFM413) and *Cosmospora vilior* (BRFM1198) acidified the medium below 3 but did not produce detectable amounts of organic acids. Therefore, the decrease in pH in our experiments cannot be explained by the sole release of large amount of organic acids. The acidification of the medium is probably mainly due to the removal of the ammonium from ammonium sulfate salt, used as nitrogen source, or excretion of H^+^ ions from the assimilation of NH_4_^+^.

### Organic acid and ethanol production in the *Basidiomycota* phylum

In the *Basidiomycota* phylum, only 6 strains out of 20 produced organic acids or ethanol. *Pycnoporus coccineus* (BRFM1396) was the only *Basidiomycota* producing several metabolites with 0.6 g.L^−1^ of gluconic acid, 0.2 g.L^−1^ of formic acid and 0.2 g.L^−1^ of ethanol (Figure 2). *Stereum hirsutum* (BRFM889), *Tinctoporellus epimiltinus* (BRFM1229) and *Fomitiporia mediterranea* (BRFM1315) produced only ethanol at concentrations between 0.12 and 0.19 g.L^−1^. Two strains: *Postia stiptica* (brown-rot, BRFM1148) and *Ganoderma weberianum* (white-rot, BRFM1548) produced only oxalic acid at 0.06 and 0.08 g.L^−1^, respectively. Generally, oxalic acid is accumulated in large quantities by brown-rot fungi and detected in lower amounts in white-rot fungi [8],[10]. This difference was attributed to the inability of brown-rot fungi to undertake an active regulation of oxalic acid concentration [10]. The other brown-rot tested, *Gloeophyllum sepiarium*, produced neither organic acids nor ethanol. Interestingly, *P. coccineus* (white-rot) produced 0.2 g.L^−1^ of formic acid. Formic acid production by this strain might be the result of oxalate decarboxylation, as described previously for white-rots [11].

### Organic acid and ethanol production in the *Ascomycota* phylum

Out of the 40 strains, 6 strains of *Ascomycota* produced neither organic acids nor ethanol at detectable level: *Cosmospora vilior* (BRFM982 and BRFM1198), *Nectria pseudocinnabarina* (BRFM1288), *Xylaria schweinitzii* (BRFM1447), *Hypomyces luteovirens* (BRFM1580), and *Cordyceps militaris* (BRFM1581). Ascorbic, fumaric and itaconic acids were detected in only a few supernatants, all from *Aspergilli,* and at concentrations below the limit of quantification (0.05 g.L^−1^). The maximal metabolite concentrations were all observed in *Aspergilli* culture media and ranged from 0.1 g.L^−1^ for lactic and tartaric acids to more than 2 g.L^−1^ for citric, formic, gluconic acids and ethanol (Table 1). Indeed, among the fungal kingdom, *Aspergilli* are well known for their ability to accumulate large amounts of organic acids [2],[18]. In our culture conditions the concentration obtained were low compared to the literature where the conversion of glucose into organic acid is described to approach 100% for some *Aspergilli* in optimized conditions [2] *.* This can be explained by the fact that accumulation of organic acid is strongly influenced by the medium composition [2],[18]. These results show that, in the *Aspergillus* genus, the major metabolite secreted is different depending on the strain.

**Table 1:** **Highest concentrations of LMWOA and ethanol obtained at day 6 of incubation, for each compound and the corresponding producing strains**

Compound	Fungal strain	g.L ^−1^
	***Ascomycota***	
**Ethanol**	*A. niger* (BRFM421)	4.1
**Gluconic acid**	*A. niger* (BRFM431)	3.7
**Formic acid**	*A. flavipes* (BRFM456)	3.3
**Citric acid**	*A. niger* (BRFM422)	2.2
**Succinic acid**	*A. flavipes* (BRFM456)	1.8
**Oxalic acid**	*A. niger* (BRFM420)	1.6
**Malic acid**	*A. niger* (BRFM103)	0.6
**Acetic acid**	*A. niger* (BRFM428)	0.4
**Propionic acid**	*A. niger* (BRFM422)	0.2
**Butyric acid**	*A. flavus* (BRFM821)	0.2
**Isobutyric acid**	*A. niger* (BRFM432)	0.2
**Tartaric acid**	*A. niger* (BRFM420)	0.1
**Lactic acid**	*A. niger* (BRFM428)	0.1
**Ascorbic acid**	*A. niger* (BRFM280)	<0.05*
**Fumaric acid**	*A. niger* (BRFM438)	<0.05*
**Itaconic acid**	*A. terreus* (BRFM111)	<0.05*
	***Basidiomycota***	
**Ethanol**	*S. hirsutum* BRFM889)	0.2
**Gluconic acid**	*P. coccineus* (BRFM1396)	0.6
**Formic acid**	*P. coccineus* (BRFM1396)	0.2
**Oxalic acid**	*G. weberianum* (BRFM1548)	0.1

*limit of quantification.

Six strains of *Aspergillus* (BRFM103, BRFM420, BRFM421, BRFM422, BRFM431 and BRFM434) have been selected for further studies due to their high organic acids or ethanol production, and to the variety of organic acids produced. *A. brasiliensis* BRFM103, *A. niger* BRFM421, and *A. niger* BRFM434 were selected for their ability to produce ethanol, 3.6, 4.1, and 2.5 g.L^−1^, respectively. *A. niger* BRFM420 was selected for its production of oxalic acid (1.6 g.L^−1^). *A. niger* BRFM422 was selected for its production of citric acid (2.2 g.L^−1^) and *A. niger* BRFM431 was selected for its production of citric (2.1 g.L^−1^) and gluconic acids (3.7 g.L^−1^). At day 6 of incubation 14.2 to 22.9 g.L^−1^ of glucose remained in the medium and these strains converted 8 to 15% of the glucose consumed to the main organic acid or ethanol (Table 2).

**Table 2:** **Concentrations and conversion yields for the 6 best organic acid producers**

	Concentration (g.L ^−1^ )	Mean Y _P/S_ (%)
***Aspergillus brasiliensis*** **(BRFM103)**		
Oxalic acid	0.4 ± 0.1	1.2
Citric acid	0.5 ± 0.1	1.5
Malic acid	0.7 ± 0.2	2.0
**Ethanol**	3.9 ± 1.0	10.8
Residual glucose	14.2 ± 1.7	
***Aspergillus niger*** **(BRFM420)**		
**Oxalic acid**	2.0 ± 0.4	7.0
Citric acid	0.5 ± 0.1	1.9
Residual glucose	21.6 ± 3.8	
***Aspergillus niger*** **(BRFM421)**		
Oxalic acid	0.4 ± 0.1	1.4
**Gluconic acid**	2.6 ± 0.5	9.5
Malic acid	0.4 ± 0.1	1.6
**Ethanol**	4.0 ± 0.7	14.5
Residual glucose	22.9 ± 1.1	
***Aspergillus niger*** **(BRFM422)**		
Oxalic acid	0.4 ± 0.1	1.3
**Citric acid**	2.5 ± 0.6	7.7
Tartaric acid	0.2 ± 0.1	0.6
**Gluconic acid**	3.4 ± 0.1	10.4
Succinic acid	0.8 ± 0.1	2.3
Fumaric acid	0.7 ± 0.2	2.3
Residual glucose	17.6 ± 1.3	
***Aspergillus niger*** **(BRFM431)**		
Oxalic acid	0.9 ± 0.1	3
**Citric acid**	2.4 ± 0.4	8.2
**Gluconic acid**	4.7 ± 0.6	15.6
Malic acid	0.4 ± 0.1	1.5
Succinic acid	0.7 ± 0.1	2.2
Residual glucose	20.4 ± 1.6	
***Aspergillus niger*** **(BRFM434)**		
Oxalic acid	0.6 ± 0.1	2.2
Citric acid	0.4 ± 0.1	1.4
**Gluconic acid**	2.4 ± 0.8	8.5
**Ethanol**	2.1 ± 0.5	7.4
Residual glucose	21.9 ± 1.7	

Yields are expressed in g of product per g of glucose consumed.

(± SD), n = 3.

In order to confirm the identity of organic acids of applied interest observed by HPLC-UV (i.e. citric, lactic, malic, and oxalic acid), supernatants from fresh cultures of *A. brasiliensis* BRFM103, *A. niger* BRFM422 and *A. niger* BRFM428 were analyzed by GC-MS. These 4 organic acids were detected in all the supernatants. With HPLC-UV, malic acid was not detected in the supernatants of strains BRFM422 and BRFM428, and lactic acid was detected only in BRFM428. This result suggests that these three strains are able to produce citric, lactic, malic and oxalic acid. However, only BRFM103 produced malic acid and BRFM422 produced lactic acid at amounts detectable by HPLC-UV. Besides, we confirm the high ethanol production by BRFM103 and found smaller amounts of ethanol in BRFM422 and BRFM428 as well. This ethanol production was also found by HPLC-RI (data not shown) showing a biological variability compared to the first cultures.

As expected the two main organic acids produced by the 6 *Aspergilli* strains were citric acid and gluconic acid [2],[18]. Interestingly, all these strains also produced oxalic acid. For most of them the production of oxalic acid was low and ranged from 0.4 to 0.9 g.L^−1^. This is consistent with the literature since oxalic acid production has been shown to be inhibited at pH below 3 by *A. niger* [27] and by ammonium and excess of substrate [1],[28]. One exception is *A. niger* BRFM420 which produced 2 g.L^−1^ of oxalic and with a conversion yield of 7 g oxalic acid/100 g of glucose consumed. For this strain, the only other organic acid detected was citric acid at 0.5 g.L^−1^. Even if this conversion yield is low compared to yields obtained in optimum conditions [29], this strain is particularly interesting since it seems more disposed than other *Aspergilli* to produce oxalic acid, even when grown in conditions not promoting the production of this organic acid.

Regarding ethanol production, the best yield, 14.5%, was observed with *A.niger* (BRFM421). *A. oryzae* and *Rhizopus oryzae* have been shown to convert 51.8% of glucose into ethanol [30]. However, a complex medium was used in this study, therefore glucose was not the sole carbon source. As a comparison, *Saccharomyces cerevisiae*, the fermentative organism used for industrial ethanol production, has a maximum theorical yield on glucose of 51.1%, and industry processes are considered economically relevant above 90% of this yield [31]. The main drawback of ethanol production from biomass using *Saccharomyces* is that the naturally occurring yeast cannot metabolize xylose, a product of biomass degradation [32]. These findings could be of interest for the production of 2nd generation ethanol from hemicelluloses and consolidated bioprocessing of biomass to ethanol [33].

## Methods

### Strains

**Figure 3:**
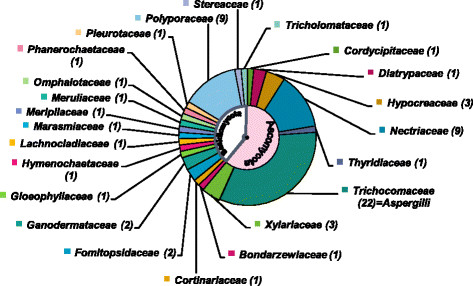
**Repartition of the strains selected for the screening in the** ***Ascomycota*** **and** ***Basidiomycota*** **phyla.** 40 strains of *Ascomycota* representing 6 families and 26 strains of Basidiomycota representing 16 families were screened.

All the strains were provided by the fungal culture collection (BRFM) of the International Centre of Microbial Resources (CIRM-CF; http://www6.inra.fr/cirm_eng/cirm-cf, Marseille, France) of the French National Institute for Agricultural Research (INRA, Marseille, France). At least one species from each family represented at the CIRM-CF was selected in order to analyze the available biodiversity. More strains and species were studied for families largely represented in the collection to achieve a better geographic diversity (Table 3, Figure 3). In total, 66 strains from 47 different species representing 23 fungal families were studied for organic acid production and other metabolic end-products. 40 of these strains originated from the *Ascomycota* phylum and 26 strains originated from the *Basidiomycota* phylum. Strains were cultivated on malt agar medium for mycelium expansion prior to inoculation of liquid cultures.

**Table 3:** **List of the strains studied and their geographic origin and corresponding BRFM numbers**

	Current name	Family	Continent	BRFM
***Ascomycota***				
	*Cordyceps militaris*	*Cordycipitaceae*	Europe	1581
	*Eutypella scoparia*	*Diatrypaceae*	Central America	1014
	*Hypocrea lixii*	*Hypocreaceae*	Europe	1058
	*Hypocrea lixii*	*Hypocreaceae*	South America	1204
	*Hypomyces luteovirens*	*Hypocreaceae*	Europe	1580
	*Cosmospora vilior*	*Nectriaceae*	Central America	982
	*Nectria pseudotrichia*	*Nectriaceae*	Central America	1017
	*Cosmospora vilior*	*Nectriaceae*	Europe	1198
	*Haematonectria haematococca*	*Nectriaceae*	South America	1214
	*Nectria pseudocinnabarina*	*Nectriaceae*	South America	1287
	*Nectria pseudocinnabarina*	*Nectriaceae*	Central America	1288
	*Lanatonectria flocculenta*	*Nectriaceae*	South America	1387
	*Haematonectria guyanensis*	*Nectriaceae*	South America	1437
	*Lasionectria lichenocola*	*Nectriaceae*	Europe	1442
	*Sinosphaeria bambusicola*	*Thyridiaceae*	NA*	1245
	*Aspergillus brasiliensis*	*Trichocomaceae*	Europe	103
	*Aspergillus flavipes*	*Trichocomaceae*	Europe	456
	*Aspergillus flavus*	*Trichocomaceae*	NA	99
	*Aspergillus flavus*	*Trichocomaceae*	Africa	821
	*Aspergillus niger*	*Trichocomaceae*	Africa	107
	*Aspergillus niger*	*Trichocomaceae*	NA	280
	*Aspergillus niger*	*Trichocomaceae*	Central America	419
	*Aspergillus niger*	*Trichocomaceae*	Central America	420
	*Aspergillus niger*	*Trichocomaceae*	Central America	421
	*Aspergillus niger*	*Trichocomaceae*	Central America	422
	*Aspergillus niger*	*Trichocomaceae*	Central America	427
	*Aspergillus niger*	*Trichocomaceae*	Central America	428
	*Aspergillus niger*	*Trichocomaceae*	Central America	431
	*Aspergillus niger*	*Trichocomaceae*	Central America	432
	*Aspergillus niger*	*Trichocomaceae*	Central America	434
	*Aspergillus niger*	*Trichocomaceae*	Central America	438
	*Aspergillus niger*	*Trichocomaceae*	Central America	439
	*Aspergillus niger*	*Trichocomaceae*	Europe	449
	*Aspergillus oryzae*	*Trichocomaceae*	Europe	488
	*Aspergillus tamarii*	*Trichocomaceae*	South America	1520
	*Aspergillus terreus*	*Trichocomaceae*	Europe	111
	*Aspergillus tubingensis*	*Trichocomaceae*	South America	1521
	*Xylaria laevis*	*Xylariaceae*	South America	1243
	*Xylaria schweinitzii*	*Xylariaceae*	South America	1447
	*Hypoxylon investiens*	*Xylariaceae*	South America	1445
***Basidiomycota***				
	*Heterobasidion annosum*	*Bondarzewiaceae*	NA	238
	*Gymnopilus junonius*	*Cortinariaceae*	Europe	969
	*Ischnoderma benzoinum*	*Fomitopsidaceae*	Europe	1133
	*Postia stiptica*	*Fomitopsidaceae*	Europe	1148
	*Amauroderma sp.*	*Ganodermataceae*	South America	1359
	*Ganoderma weberianum*	*Ganodermataceae*	South America	1548
	*Gloeophyllum sepiarium*	*Gloeophyllaceae*	Europe	988
	*Fomitiporia mediterranea*	*Hymenochaetaceae*	Europe	1315
	*Dichostereum effuscatum*	*Lachnocladiaceae*	Europe	91
	*Lentinula edodes*	*Marasmiaceae*	NA	353
	*Grifola frondosa*	*Meripilaceae*	Europe	1162
	*Abortiporus biennis*	*Meruliaceae*	Europe	1215
	*Omphalotus olearius*	*Omphalotaceae*	Europe	1195
	*Phanerochaete chrysosporium*	*Phanerochaetaceae*	Europe	413
	*Pleurotus ostreatus*	*Pleurotaceae*	Europe	1326
	*Grammothele fuligo*	*Polyporaceae*	South America	1046
	*Daedaleopsis confragosa*	*Polyporaceae*	Europe	1187
	*Perenniporia ochroleuca*	*Polyporaceae*	Europe	1192
	*Earliella scabrosa*	*Polyporaceae*	South America	1220
	*Tinctoporellus epimiltinus*	*Polyporaceae*	South America	1229
	*Trametes menziesii*	*Polyporaceae*	Oceania	1281
	*Trametes sp.*	*Polyporaceae*	South America	1361
	*Pycnoporus coccineus*	*Polyporaceae*	Oceania	1396
	*Polyporus squamosus*	*Polyporaceae*	Europe	1531
	*Stereum hirsutum*	*Stereaceae*	Europe	889
	*Panellus serotinus*	*Tricholomataceae*	Europe	1284

Families are sorted in alphabetical order, when several strains from one family were tested; species were sorted in alphabetical order and by increasing BRFM number for strains from the same species.

*NA: not available.

### Chemicals and reagents

Ultrapure water (conductivity 18.2 mΩ) was used in all experiments. For fungal cultures, malt extract was purchased from VWR, EDTA-Na_2_ and glucose were from Sigma, (NH_4_)_2_SO_4_ and CaCl_2_ were from Panreac, H_3_BO_3_, MnCl_2_, FeSO_4_, CuSO_4_, CoCl_2_ Na_2_MoO_4_, MgSO_4,_ KH_2_PO_4_, and ZnSO_4_ were purchased from Prolabo and Bacto agar was purchased from Fischer.

For analytic methods, CD_3_OH, HPLC grade organic acid standards (acetic, adipic acid, L-ascorbic acid, benzoic acid, butyric acid, citric acid, isobutyric acid, formic acid, fumaric acid, L-(+)-lactic acid, DL-isocitric acid trisodium salt hydrate, maleic acid, malonic acid, D-(+)-malic acid, oxalic acid, phytic acid, propionic acid, (−)quinic, succinic acid, shikimic acid,D-(−)-tartaric acid), methylchloroformate (MCF), and glucose were purchased from Sigma, HPLC grade ethanol was purchased from Fluka. Dichloromethane was from Carlo Erba Reagents.

### Liquid cultures

The liquid medium was composed of (NH_4_)_2_SO_4_ (8 mM), CaCl_2_ (1 mM), KH_2_PO_4_ (11 mM), MgSO_4_ (2 mM), glucose 5% (wt/vol) and trace elements and had an initial pH of 5.5. The final concentrations of trace elements was ZnSO_4_ (76 μM), H_3_BO_3_ (178 μM), MnCl_2_ (25 μM), FeSO_4_ (18 μM), CoCl_2_ (7.1 μM), CuSO_4_ (6.4.μM), Na_2_MoO_4_ (6.2 μM), and EDTA-Na_2_ (174 μM). The glucose concentration had to be high, in order to be close to industrial conditions for organic acid production in *Ascomycota* [34]. In previous screenings of Basidiomycota for organic acid production, the glucose concentration was set to 5% [12]. This concentration was therefore chosen to be suitable for both *Ascomycota* and *Basidiomycota* growth. For *Basidiomycota*, which hardly grow on such media, Tatum vitamins [35] and yeast extract (0.03 g.L^−1^) were added to the liquid medium. Cultivations were carried out in 250 mL baffled flasks to facilitate oxygen transfert. They contained 100 mL of liquid medium and were incubated for 6 days at 30°C in an orbital incubator at 120 and 140 rpm for *Ascomycota* and *Basidiomycota*, respectively. For *Aspergilli*, the liquid medium was inoculated at an initial titer of 2 × 10^6^ spores.mL^−1^. For other fungi (*Basidiomycota* and other *Ascomycota*), that do not produce enough spores in our growth conditions, fungal disks, 4 mm in diameter, were collected from the solid medium. For each strain, 3 tubes containing 1 fungal disk and 1 mL sterile water were crushed at a frequency of 4 s^−1^ during 60 s using a FastPrep-24 (MPBio, Solon, OH, USA). The 3 tubes were then mixed together. Afterwards, 1 mL of the inoculum preparation was added to each flask. Cultures were carried out in triplicates.

### Analytical methods

During the incubation, the acidity of the culture media was evaluated daily with pH paper (Duotest®, Machery-Nagel, Düren, Germany). At days 3 and 6 of incubation, 2 mL samples were harvested from the media. Mycelium was removed by centrifugation and the supernatant was collected after ultra-filtered using Vivaspin® 5kD (VWR, Strasbourg, France) tubes to remove proteins. The filtrates were analyzed for organic acids by HPLC (Agilent 1100 series HPLC, Santa Clara, CA, USA) using an Aminex HPX-87H organic acid analysis column (100 mm × 7.8 mm, Biorad, Marnes-la-Coquette, France). The column was equilibrated in 2.5 mM H_2_SO_4_ at 35°C and samples were eluted with 2.5 mM H_2_SO_4_ at a 0.6 mL.min^−1^ flow rate. Organic acids were detected with a UV detector at 210 nm (G1314A, Agilent HPLC 1100 series) and ethanol and glucose were detected with a differential refractometer (HP1047A, Hewlett Packard). Data were acquired with ChemStation software (Agilent, Hewlett Packard, Waldbronn, Germany). The first analysis of metabolite secretion was performed with pools of the three replicates of each strain in order to get an estimation of the mean organic acids production. The supernatants of the higher producers were then analyzed separately and the identification of organic acids and ethanol was further studied using GC-MS.

Supernatants of the cultures were harvested by centrifugation and organic acids were directly derivatized, without ultrafiltration, using MCF as previously described [36], with some modifications. Briefly, 190 μL of the supernatants were directly alkalinized with 10 μL NaOH 2.5 M and derivatized by two consecutive additions of 20 μL MCF. After derivatization, the methylated compounds were extracted with dichloromethane. GC-MS analysis of organic acids was performed with an Agilent 5973 N system, equipped with an Omegawax (Supelco, Bellefont, PA, USA), 30 m × 250 μm i.d. × 0,25 μm thick films. The carrier gas was helium at 35 cm.s^−1^. The oven program temperature started at 40°C during 3 min, then rose at 8°C per minute to 230°C and held at this temperature for 15 min. 2 μL of extract were injected in split injector port with split ratio of 10. Mass spectra in the 29 to 400 m/z range were recorded at a scanning speed of 2 scans.s^−1^ and an electronic ionization at 70 eV. Compounds were identified by matching compound mass spectra to the NIST library and using pure authentic chemical standards for each organic acid studied, derivatized using the same process as biological samples.

Ethanol concentration was determined in 2 mL supernatants by Head-space-GC-MS with addition of 0.4 mL of the internal standard CD_3_OH at 0.6 g.L^−1^. A calibration curve was prepared with 201.8, 403.6 and 807.2 μg of ethanol with CD_3_OH as internal standard as above. For GC we used the GC–MS QP2010 Shimadzu with capillary column Cp_wax_52cb 30 m × 0.32 mm × 0.5 μm (Varian, Inc, Palo Alto, USA) equipped with an autosampler AOC5000. The sealed vials were placed at 50°C for 8 min with 500 rpm shaking before 0.5 mL of the headspace were drawn out with a gas syringe heated at 60°C and injected with in a split injector with a split ratio of 10. The carrier gas was helium at 35 cm.s^−1^ and oven temperature was isothermal at 50°C. Mass detector conditions were: electronic impact ionization mode (70 eV), temperature of source 200°C with data collected using SIM for selected ions m/z 45/46 and 35/30 for ethanol and CD_3_OH respectively.

## Conclusion

The potentiality of a wide panel of fungus for organic acids production has been studied in glucose based liquid media at acidic pH to take into account industrial up- and down-stream technical and economical issues. Strains were sorted in two clusters considering their organic acid and ethanol production at day 6 of incubation, showing that some strains, even from the same species, seem to have particular predispositions for some metabolites.

Among 26 *Basidiomycota* tested, only two: *Postia stiptica* (brown-rot) and *Ganoderma weberianum* (white-rot) produced oxalic acid. Ethanol is the common metabolite in the fungal kingdom, regardless the geographic origin of the strains, but with different extent depending on the strain. Although yeasts have very competitive ethanol productivity on simple sugars, our best ethanol producers may be good candidates for consolidated bioprocessing (CBP) of cellulosic biomass for second generation ethanol production.

Among the *Ascomycota, Aspergilli* clearly make a distinct cluster for their various and high concentration organic acid production; this illustrates the relevance of organic acids in the chemotaxonomy of *Aspergilli*. Some of these strains showed particular ability to produce malic, oxalic, gluconic and citric acids or ethanol at low pH. This production could be further improved by genetic modifications. The high intra-specific variability in metabolite production stresses the importance of screenings for a good choice of studied strains.

This study provides a better knowledge of the capability of filamentous fungi to produce organic acids which should allow a greater exploitation of filamentous fungi in synthetic biology, metabolic studies and industrial exploitation of organic acids.

## Authors’ original submitted files for images

Below are the links to the authors’ original submitted files for images. Authors’ original file for figure 1
Authors’ original file for figure 2
Authors’ original file for figure 3

## Abbreviations

CIRM-CF: Fungal culture collection of the international centre of microbial resources
MCF: Methyl chloroformate
