# Supplementary figures and images for: Exploring fungal biodiversity: organic acid production by 66 strains of filamentous fungi
Source: Fungal Biol Biotechnol. 2014 Nov 1;1:1. doi: 10.1186/s40694-014-0001-z (PMC4599204; doi:10.1186/s40694-014-0001-z)

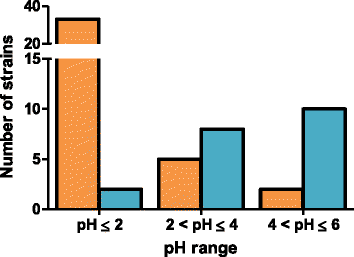

Supplement: Supplementary file 1 — Authors’ original file for figure 1 [file 40694_2014_1_MOESM1_ESM.gif]

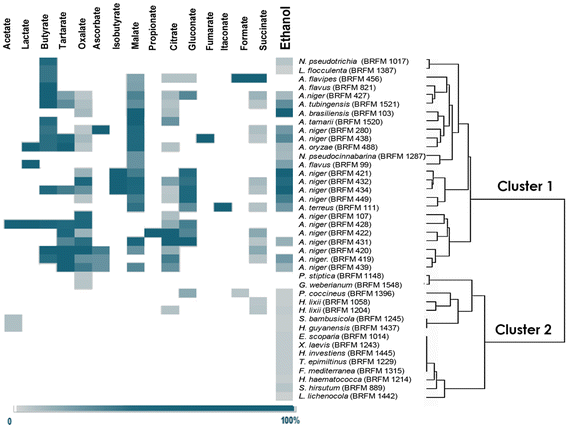

Supplement: Supplementary file 2 — Authors’ original file for figure 2 [file 40694_2014_1_MOESM2_ESM.gif]

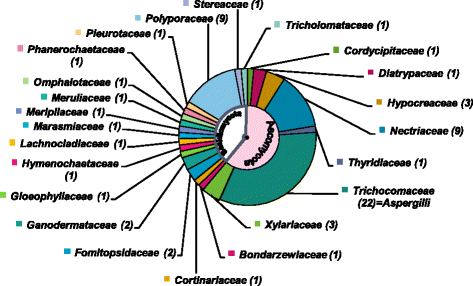

Supplement: Supplementary file 3 — Authors’ original file for figure 3 [file 40694_2014_1_MOESM3_ESM.gif]
